# Global burden of trauma: Need for effective fracture therapies

**DOI:** 10.4103/0019-5413.50843

**Published:** 2009

**Authors:** George Mathew, Beate P Hanson

**Affiliations:** Division of Orthopaedic Surgery, Department of Surgery, McMaster University, Ontario, Canada and AO Clinical Investigation, Zurich, Switzerland

**Keywords:** Trauma, fracture, bone stimulation therapies, orthopedic community

## Abstract

Orthopedic trauma care and fracture management have advanced significantly over the last 50 years. New developments in the biology and biomechanics of the musculoskeletal system, fixation devices, and soft tissue management have greatly influenced our ability to care for musculoskeletal injuries. Many therapies and treatment modalities have the potential to transform future orthopedic treatment by decreasing invasive procedures and providing shorter healing times. Promising results in experimental models have led to an increase in clinical application of these therapies in human subjects. However, for many modalities, precise clinical indications, timing, dosage, and mode of action still need to be clearly defined. In order to further develop fracture management strategies, predict outcomes and improve clinical application of newer technologies, further research studies are needed. Together with evolving new therapies, the strategies to improve fracture care should focus on cost effectiveness. This is a great opportunity for the global orthopedic community, in association with other stakeholders, to address the many barriers to the delivery of safe, timely, and effective care for patients with musculoskeletal injuries in developing countries.

## INTRODUCTION

The global burden of injury is staggering, and injuries are predicted to be a leading cause of death and disability over the next few decades.^1^–^9^ In 2001, injuries in developing countries accounted for 11% of the world's disease burden, and ranked 11th in all causes for both mortality and morbidity.^2^ It also is estimated that in the developing countries 6 million will die and 60 million will be injured, or disabled, in the next 10 years.^7^ With fractures accounting for the majority of trauma in developing nations, novel therapies are desperately needed to optimize patient outcomes.

Orthopedic trauma care and fracture management have seen significant advances over the last 50 years. New developments in the biology and biomechanics of the musculoskeletal system, fixation devices, and soft tissue management have greatly influenced our ability to care for musculoskeletal injuries. Many therapies and treatment modalities have the potential to transform future orthopedic treatment by decreasing invasive procedures and providing shorter healing times. In order to further develop fracture management strategies, predict outcomes, and improve clinical application of newer technologies, further research studies are needed. Together with evolving new therapies, the strategies to improve fracture care should focus on cost effectiveness. This is a great opportunity for the global orthopedic community, in association with other stakeholders, to address the many barriers to the delivery of safe, timely, and effective care for patients with musculoskeletal injuries in developing countries.

## FRACTURE OUTCOMES: CURRENT CHALLENGES

The biology of fracture healing is an organized and complex process that restores skeletal integrity by reconstitution of bone. Although fracture healing is a consistent and reliable biological response, its failure can lead to devastating clinical consequences. It is estimated that delayed or impaired healing will occur in 5–10% of the 5.6 million fractures that occur annually in the United States, and up to 10% of all fractures will require additional surgical procedures for impaired healing.^10^ Furthermore, a recent literature from the United States suggests that the lifetime risk of fracture is 50% for males and 33% for females.^11^

The standard treatment in developed countries for delayed healing and nonunions has been open surgical fixation with autogenous bone grafting. This method provides the essential elements for bone regeneration: osteoinduction, osteoconduction, and osteoprogenitor cells. However, a frequent problem associated with autogenous bone grafting is donor site morbidity.^12^ Currently, many other biological and biophysical approaches are available to minimize the occurrence of delayed unions and nonunions. The biological approaches include gene therapy, tissue engineering, osteoconductive biomaterials, growth factors, bone-marrow aspirates, and osteocompetent cells. Mechanical stimulation by low-intensity ultrasound, electromagnetic fields, and extracorporeal shock-wave therapy are some of the biophysical approaches available. Recent studies have also looked at the impact of drugs and hormonal therapy, especially parathyroid hormone, on bone repair.^13^

## BIOLOGICAL METHODS TO FACILITATE BONE HEALING AND REPAIR

Induction of bone healing by chemical, biophysical, and hormonal means is a rapidly growing area. Osteoconductive materials act as a scaffold and support new bone formation through in-growth of the host bone.^14^–^17^ There are several types of osteoconductive agents that can be used to heal fractures. Calcium sulfates and phosphates are strong in compression but weak in tension and shear.^18^,^19^ Hydroxyapatite and tricalcium phosphate are examples of calcium phosphates with osteoconductive and osteointegrative properties.^16^,^17^ Calcium sulfate has predictable resorption *in vivo* due to minimal trace elements and biodegradability whereas tricalcium phosphate is more rapidly absorbed than hydroxyapatite due to increased porosity and solubility.^14^ Due to these properties, tricalcium phosphate is inadequate when structural support is required. Type I collagen is another osteoconductive material and is the most abundant extracellular bone-matrix protein. Type I collagen by itself is a poor graft substitute but when combined with bone morphogenetic proteins, osteoprogenitor precursors or hydroxyapatite, its osteoconductive potential increases considerably.^17^ Bioactive glass, biodegradable polymers, and porous and plasma-coated metals are other examples of osteoconductive materials, but all of these materials need further studies to analyze their clinical effectiveness.^16^

Several randomized studies of osteoconductive materials have shown promising results. Shors compared coralline hydroxyapatite to an autograft for 174 long-bone defects and found that time to union was not significantly different at 4.5 months.^19^ Bucholz *et al*. in an earlier study of coralline hydroxyapatite in tibial plateau fractures reported similar results.^20^ Mattsson and Larsson compared closed reduction and cannulated screw fixation versus cannulated screws plus calcium phosphate cement in the treatment of displaced fractures of the femoral neck and found no difference in pain and muscle strength between groups, but 34 patients (14 in the control group and 20 in the calcium phosphate group) required conversion to total hip replacement.^21^ Mattsson *et al*. also reported favorable outcomes with the use of calcium phosphate cement in unstable peritrochanteric and displaced fractures of the neck of femur.^22^,^25^ Several authors have reported favorable results with the use of calcium phosphate cement.^26^,^27^ Chapman *et al*. compared the use of iliac bone graft to a collagen composite for long-bone fractures and found no difference in outcomes except for a higher rate of infection with the use of iliac bone graft.^28^ Cornell *et al*. reported similar results and found no significant difference in hospital length of stay or pain scores.^29^

Various cell types, growth receptors, and growth factors are present within a fracture callus and modulation of these growth factors is postulated to positively influence bone healing.^30^–^33^ Research efforts have focused on the development of appropriate carriers or delivery systems for growth factors in order to effectively deliver them to the fracture site. Type I collagen, synthetic polymers and hyaluronic acid gels are examples of such agents.^34^–^37^ Unlike bone morphogenetic proteins (BMPs) which are proven in several human clinical trials as a potent osteoconductive material, certain other growth factors, such as transforming growth factor-beta, insulin-like growth factor, and platelet-derived growth factor-beta, have insufficient preclinical data to determine efficacy.^38^–^52^ Friedlaender *et al*. reported that using recombinant human morphogenetic protein-7 (rhBMP-7) was as good as bone grafting and was not associated with donor site morbidity that usually occurs in more than 20% of patients receiving an autograft.^53^ The BMP-2 Evaluation in Surgery for Tibial Trauma (BESTT) Study Group compared the effects of two different exogenous rhBMP-2 concentrations versus no rhBMP-2 on the healing of nailed open tibial fractures.^47^ This group reported a dose-dependent effect, with faster healing times and fewer secondary interventions for delayed unions and nonunions. Several Level-I studies of rhBMPs have shown promising results as well.^48^–^52^ Further studies are needed to determine appropriate dosages, optimum time, and modes of delivery, duration of treatment, and precise clinical indications for use. It is also important to determine a cost-to-benefit analysis for the use of these agents.

Several studies have looked at the role of percutaneous bone-marrow aspirates in the closed treatment of fractures, although, currently no Level-I study has directly compared the efficacy of bone-marrow aspirates to autologous bone graft. Although little information is available regarding the number and concentration of cells that are necessary for bone repair, several studies have reported on the human variability with respect to bone-marrow cellularity and osteoblast progenitor-cell prevalence.^54^–^59^ Further randomized studies examining the growth factor use and its combination with collagen to increase stem-cell proliferation and differentiation are needed.^60^

Demineralized bone matrix is an osteoconductive agent that provides no structural support but is used for filling defects and cavities.^61^,^62^ They are produced by acid extraction of bone and contain noncollagenous proteins, growth factors, BMPs and type I collagen.^63^ Demineralized bone matrix is available in various formulations: freeze-dried powder, granules, strips, gel, chips, or calcium sulphate granules.^61^,^62^ The efficacy of these products ranges widely, depending on bone type, sterilization process, carrier, quantity of BMPs present, and ratios of each.^64^,^65^ The osteoinductive efficacy of demineralized bone matrix has been determined in animal studies but, currently no randomized studies have been done on humans comparing the effects of demineralized bone matrix with that of the autologous bone graft.^63^ The current recommendation for the use of demineralized bone matrix is as graft extenders.^61^,^63^

Platelet gels have generated a considerable interest in the recent years. These are made by isolating a concentration of platelets from the patient's own blood. Platelet gels contain growth factors and function as osteoinductive agents. They can thus play a key role in bone formation and maturation of osseous fusions.^66^,^67^ Current indications for the use of platelet gels are as graft extenders. Further studies are needed to assess the optimum concentration of the different factors and to determine which factors would provide the greatest effect.

Human gene therapy for fracture repair is another attractive option due to the decreased invasiveness of the technique. However, presently there are no Level-I studies to inform clinical decision making regarding bone repair. This therapy relies on treating human diseases by transferring genetic material to individual cells,^69^ thus re-establishing damaged cellular function, introducing a new function, and/or interfering with an existing function.^70^ Three fundamental^68^ steps critical for the success of gene therapy are identification of the specific genetic material to be transferred, the method of transfer, and the cell type that would incorporate the material.^71^,^72^ Encouraging preclinical data are available on tissue repair, cancer, and regeneration of bone cartilage, ligament, tendon, meniscus, and intervertebral disk.^71^–^74^ This technique has also been applied to other areas including osteoporosis, aseptic loosening, genetic diseases, musculoskeletal infections, and tumors.^74^–^77^ Spinal fusions and repair of segmental defects of long bones have seen impressive preclinical study results.^71^,^73^,^74^

Orthopedic tissue engineering is still in experimental stages and combines the use of three-dimensional scaffold materials, cells, and release of growth factors.^78^ The goal in tissue engineering is reconstitution of tissues that have failed to regenerate or heal spontaneously. Pleuripotent mesenchymal stem cells are capable of differentiating into bone-forming osteoblasts with appropriate growth factors present both *in vitro* and *in vivo*.^79^,^80^ These cells are biopsied from the patient and are grown on engineered biomaterials *in vitro* and implanted at the desired treatment location.^81^ Many novel substances like biodegradable polymers and ceramics with adsorbed growth factors are in trial as substitutes for skeletal elements such as cartilage and bones.^82^–^85^

Intermittent exposure to parathyroid hormone may benefit fracture healing and implant fixation, as seen in preclinical studies.^86^–^88^ Continuous exposure to parathyroid hormone results in bone resorption, but intermittent doses result in bone formation through an increased osteoblastic activity.^88^,^89^ Further clinical studies are needed to determine if this hormone will benefit patients with fractures, what the optimal treatment duration might be, and if resorption inhibitor therapy after parathyroid hormone treatment will improve fracture healing.^88^,^89^

## CONCLUSIONS

Musculoskeletal injuries are a substantial burden in developing countries such as India; the problem is complex, multidimensional, and can only be solved through a multidisciplinary, multisectoral effort. While recognizing the importance of prevention, improving treatment is essential. Many therapies and treatment modalities have the potential to transform future orthopedic treatment by decreasing invasive procedures and providing shorter healing times. In order to further develop fracture management strategies, predict outcomes, and improve clinical application of newer technologies, further research studies are needed.

## References

[1] Murray CJL, Lopez AD (1996). The global burden of disease: a comprehensive assessment of mortality and disability from diseases, injuries and risk factors in 1990 and projected to 2020.

[2] Lopez AD, Mathers CD, Ezzati M, Jamison DT, Murray CJL (2006). Global burden of disease and risk factors.

[3] Mathers CD, Loncar D (2006). Projections of global mortality and burden of disease from 2002 to 2030. PLoS Med.

[4] Peden M, McGee K, Sharma G (2002). The injury chart book: a graphical overview of the global burden of injuries.

[5] Krug EG, Sharma GK, Lozano R (2000). The global burden of injuries. Am J Public Health.

[6] Norton R, Hyder AA, Bishai D, Peden M, Jamison DT, Breman JG, Measham AR, Alleyne G, Claeson M, Evans DB, Jha P, Mills A, Musgrove P (2006). Unintentional injuries. Disease control priorities in developing countries.

[7] Gururaj G (2005). Injuries in India: National Perspective Burden of Disease in India. National Commission on Macroeconomics and Health.

[8] Joshipura MK, Shah HS, Patel PR, Divatia PA, Desai PM (2003). Trauma care systems in India. Injury.

[9] Sethi AK, Tyagi A (2001). Trauma untamed as yet. Trauma Care.

[10] Wegman F (1996). Road accidents: worldwide a problem that can be tackled successfully!.

[11] Brinker MR, O'Connor DP (2004). The incidence of fractures and dislocations referred for orthopaedic services in a capitated population. J Bone Joint Surg Am.

[12] Damien CJ, Parsons JR (1991). Bone graft and bone graft substitutes: a review of current technology and applications. J Appl Biomater.

[13] Novicoff WM, Manaswi A, Hogan MV, Brubaker SM, Mihalko WM, Saleh KJ (2008). Critical analysis of the evidence for current technologies in bone-healing and repair. J Bone Joint Surg Am.

[14] Boyan B, McMillan J, Lohmann CH, Ranly DM, Schwartz Z, Laurencin CT (2003). Bone graft substitutes: basic information for successful clinical use with special focus on synthetic graft substitutes. Bone graft substitutes.

[15] Moore WR, Graves SE, Bain GI (2001). Synthetic bone graft substitutes. ANZ J Surg.

[16] Parikh SN (2002). Bone graft substitutes in modern orthopaedics. Orthopaedics.

[17] Finkemeier CG (2002). Bone-grafting and bone graft substitutes. J Bone Joint Surg Am.

[18] LeGeros RZ (2002). Properties of osteoconductive biomaterials: calcium phosphates. Clin Orthop Relat Res.

[19] Shors EC, Laurencin CT (2003). The development of coralline porous ceramic graft substitutes. Bone graft substitutes.

[20] Bucholz RW (2002). Nonallograft osteoconductive bone graft substitutes. Clin Orthop Relat Res.

[21] Mattsson P, Larsson S (2006). Calcium phosphate cement for augmentation did not improve results after internal fixation of displaced femoral neck fractures: a randomized study of 118 patients. Acta Orthop.

[22] Mattsson P, Alberts A, Dahlberg G, Sohlman M, Hyldahl HC, Larsson S (2006). Resorbable cement for the augmentation of internally-fixed unstable trochanteric fractures. A prospective, randomized, multicentre study. J Bone Joint Surg Br.

[23] Mattsson P, Larsson S (2004). Unstable trochanteric fractures augmented with calcium phosphate cement. A prospective randomized study using radiostereometry to measure fracture stability. Scand J Surg.

[24] Mattsson P, Larsson S (2003). Stability of internally fixed femoral neck fractures augmented with resorbable cement. A prospective randomized study using radiostereometry. Scand J Surg.

[25] Cassidy C, Jupiter JB, Cohen M, Delli-Santi M, Fennell C, Leinberry C, Husband J, Ladd A, Seitz WR, Constanz B (2003). Norian SRS cement compared with conventional fixation in distal radius fractures. A randomized study. J Bone Joint Surg Am.

[26] Zimmermann R, Gabl M, Lutz M, Angermann P, Gschwentner M, Pechlaner S (2003). Injectable calcium phosphate bone cement Norian SRS for the treatment of intraarticular compression fractures of the distal radius in osteoporotic women. Arch Orthop Trauma Surg.

[27] Sanchez-Sotelo J, Munuera L, Madero R (2000). Treatment of fractures of the distal radius with a remodellable bone cement: a prospective, randomized study using Norian SRS. J Bone Joint Surg Br.

[28] Chapman MW, Bucholz R, Cornell C (1997). Treatment of acute fractures with a collagen-calcium phosphate graft material. A randomized clinical trial. J Bone Joint Surg Am.

[29] Cornell CN, Lane JM, Chapman M, Merkow R, Seligson D, Henry S (1991). Multicenter trial of Collagraft as bone graft substitute. J Orthop Trauma.

[30] Trippel SB (1997). Growth factors as therapeutic agents. Instr Course Lect.

[31] Barnes GL, Kostenuik PJ, Gerstenfeld LC, Einhorn TA (1999). Growth factor regulation of fracture repair. J Bone Miner Res.

[32] Einhorn TA (1995). Enhancement of fracture-healing. J Bone Joint Surg Am.

[33] Lieberman JR, Daluiski A, Einhorn TA (2002). The role of growth factors in the repair of bone. Biology and clinical applications. J Bone Joint Surg Am.

[34] Yasko AW, Lane JM, Fellinger EJ, Rosen V, Wozney JM, Wang EA (1992). The healing of segmental bone defects, induced by recombinant human bone morphogenetic protein (rh-BMP-2). A radiological, histological, and biomechanical study in rats. J Bone Joint Surg Am.

[35] Bostrom M, Lane JM, Tomin E, Browne M, Berberian W, Turek T, Smith J, Wozney J, Schildhauer T (1996). Use of bone morphogenetic protein-2 in the rabbit ulnar non-union model. Clin Orthop Relat Res.

[36] Radomsky ML, Thompson AY, Spiro RC, Poser JW (1998). Potential role of fibroblast growth factor in enhancement of fracture healing. Clin Orthop Relat Res.

[37] Radomsky ML, Aufdermorte TB, Swain LD, Fox WC, Spiro RC, Poser JW (1999). Novel formulation of fibroblast growth factor-2 in a hyaluronan gel accelerates fracture healing in nonhuman primates. J Orthop Res.

[38] Simpson AH, Mills H, Noble B (2006). The role of growth factors and related agents in accelerating fracture healing. J Bone Joint Surg Br.

[39] Broderick E, Infanger S, Turner TM, Sumner DR (2005). Depressed bone mineralization following high dose TGF-beta-1 application in an orthopaedic implant model. Calcif Tissue Int.

[40] Schmidmaier G, Wildemann B, Gabelein T, Heeger J, Kandziora F, Haas NP, Raschke M (2003). Synergistic effect of IGF-I and TGF-beta 1 on fracture healing in rats: single versus combined application of IGF-I and TGF-beta 1. Acta Orthop Scand.

[41] Deckers MM, van Bezooijen RL, van der Horst G, Hoogendaam J, van Der Bent C, Papapoulos SE, Lowik CW (2002). Bone morphogenetic proteins stimulate angiogenesis through oeteoblast-derived vascular endothelial growth factor A. Endocrinology.

[42] Kelpke SS, Zinn KR, Rue LW, Thompson JA (2004). Site specific delivery of acidic fibroblast growth factor stimulates angiogenic and osteogenic responses *in vivo*. J Biomed Mater Res.

[43] Bail HJ, Kolbeck S, Krummrey G, Schmidmaier G, Haas NP, Raschke MJ (2002). Systemic application of growth hormone for enhancement of secondary and intramembranous fracture healing. Horm Res.

[44] Kolbeck S, Bail H, Schmidmaier G, Alquiza M, Raun K, Kappelgard A (2003). Homologous growth hormone accelerates bone healing: a biomechanical and histological study. Bone.

[45] Nash TJ, Howlett CR, Martin C, Steele J, Johnson KA, Hicklin DJ (1994). Effect of platelet-derived growth factor on tibial osteotomies in rabbits. Bone.

[46] Friedlaender GE, Perry CR, Cole JD, Cook SD, Cierny G, Muschler GF (2001). Osteogenic protein-1 (bone morphogenetic protein-7) in the treatment of tibial nonunions. J Bone Joint Surg Am.

[47] Govender S, Csimma C, Genant HK, Valentin-Opran A, Amit Y, Arbel R (2002). Recombinant human bone morphogenetic protein-2 for treatment of open tibial fractures: a prospective, controlled, randomized study of four hundred and fifty patients. J Bone Joint Surg Am.

[48] Swiontkowski MF, Aro HT, Donell S, Esterhai JL, Goulet J, Jones A (2006). Recombinant morphogenetic protein-2 in open tibial fractures. A subgroup analysis of data combined from two prospective randomized studies. J Bone Joint Surg Am.

[49] Giannoudis PV, Tzioupis C (2005). Clinical applications of BMP-7: the UK perspective. Injury.

[50] Zimmermann G, Moghaddam A, Wagner C, Vock B, Wentzensen A (2006). Clinical experience with bone morphogenetic protein 7 (BMP 7) in nonunions of long bones. Unfallchirug.

[51] Bilic R, Simic P, Jelic M, Stern-Padovan R, Dodig D, van Meerdervoort HP (2006). Osteogenic protein-1 (BMP-7) accelerates healing of scaphoid non-union with proximal pole sclerosis. Int Orthop.

[52] Jones AL, Bucholz RW, Bosse MJ, Mirza SK, Lyon TR, Webb LX (2006). BMP-2 Evaluation in Surgery for Tibial Trauma-Allograft (BESTT-ALL) Study Group. Recombinant human BMP- and allograft compared with autogenous bone graft for reconstruction of tibial diaphyseal fractures with cortical defects. J Bone Joint Surg Am.

[53] Friedlaender GE, Perry CR, Cole JD, Cook SD, Cierny G, Muschler GF (2001). Osteogenic protein-1 (bone morphogenetic protein-7) in the treatment of tibial nonunions. J Bone Joint Surg Am.

[54] Connolly J, Guse R, Lippeillo L, Dehne R (1989). Development of an osteogenic bone-marrow preparation. J Bone Joint Surg Am.

[55] Connolly JF, Guse R, Tiedemann J, Dehne R (1991). Autologous marrow injection as a substitute for operative grafting of tibial non unions. Clin Orthop Relat Res.

[56] Muschler GF, Boehm C, Easley K (1997). Aspiration to obtain osteoblast progenitor cells from human bone marrow: the influence of aspiration volume. J Bone Joint Surg Am.

[57] Hernigou P, Poignard A, Beaujean F, Rouard H (2005). Percutaneous autologous bone-marrow grafting for nonunions. Influence of the number and concentration of progenitor cells. J Bone Joint Surg Am.

[58] Hernigou P, Mathieu G, Poignard A, Manicom O, Beaujean F, Rouard H (2006). Percutaneous autologous bone-marrow grafting for nonunions. Surgical technique. J Bone Joint Surg Am.

[59] Tiedemann JJ, Connolly JF, Strates BS, Lippiello L (1991). Treatment of nonunion by percutaneous injection of bone marrow and demineralized bone matrix. An experimental study in dogs. Clin Orthop Relat Res.

[60] Cornell CN, Lane JM, Chapman M, Merkow R, Seligson D, Henry S (1991). Multicenter trial of Collagraft as bone graft substitute. J Orthop Trauma.

[61] Ferreira SD, Dernell WS, Powers BE, Schochet RA, Kuntz CA, Withrow SJ (2001). Effect of gas-plasma sterilization on the osteoinductive capacity of demineralized bone matrix. Clin Orthop Relat Res.

[62] Bae HW, Zhao L, Kanim LE, Wong P, Delamarter RB, Dawson EG (2006). Intervariability and intravariability of bone morphogenetic proteins in commercially available demineralized bone matrix products. Spine.

[63] Russell JL, Block JE (2004). Clinical utility of demineralized bone matrix for osseous defects, arthrodesis, and reconstruction: impact of processing techniques and study methodology. Orthopaedics.

[64] Carpenter JF, Pikal MJ, Chang BS, Randolph TW (1997). Rational design of stable lyophilized protein formulations: some practical advice. Pharm Res.

[65] Martin GJ, Boden SD, Titus L, Scarborough NL (1999). New formulations of demineralized bone matrix as a more effective graft alternative in experimental posterolateral lumbar spine arthrodesis. Spine.

[66] Flamini S, Francesco A, Colafarina O, Cerulli Mariani P, Pizzoferrato R, Rughetti A (2007). Autologous platelet gel for delayed union of tibial shaft treated with intramedullary nailing. Ital J Orthop Traumatol.

[67] Savarino L, Cenni E, Tarabusi C, Dallari D, Stagni C, Cenacchi A (2006). Evaluation of bone healing enhancement by lyophilized bone grafts supplements with platelet gel: a standardized methodology in patients with tibial osteotomy for genu varus. J Biomed Mater Res B Appl Biomater.

[68] Evans CH, Ghivizzani SC, Herndon JH, Wasko MC, Reinecke J, Wehling P, Robbins PD (2000). Clinical trials in gene therapy of arthritis. Clin Orthop Relat Res.

[69] Evans CH, Ghivizzani SC, Robbins PD (2004). Orthopaedic gene therapy. Clin Orthop Relat Res.

[70] Evans CH, Ghivizzani SC, Herndon JH, Robbins PD (2005). Gene therapy for the treatment of musculoskeletal diseases. J Am Acad Orthop Surg.

[71] Giannoudis PV, Tzioupis CC, Tsiridis E (2006). Gene therapy in orthopaedics. Injury.

[72] Ivkovic A, Pascher A, Hudetz D, Jelic M, Haspl M, Windhager R, Pecina M (2006). Current concepts in gene therapy of the musculoskeletal system. Acta Chir Orthop Traumatol Czech.

[73] Phillips FM, Blot PM, He TC, Haydon RC (2005). Gene therapy for spinal fusion. Spine J.

[74] Egermann M, Schneider E, Evans CH, Baltzer AW (2005). The potential for gene therapy for fracture healing in osteoporosis. Osteoporos Int.

[75] Shen W, Li Y, Huard J (2005). Musculoskeletal gene therapy and its potential use in the treatment of complicated musculoskeletal infection. Infect Dis Clin North Am.

[76] Vervoordeldonk MJ, Tak PP (2001). Gene therapy in rheumatic diseases. Best Pract Res Clin Rheumatol.

[77] Muschler GF, Nakamoto C, Griffith LG (2004). Engineering principles of clinical cell-based tissue engineering. J Bone Joint Surg Am.

[78] Pountos I, Jones E, Tzioupis C, McGonagle D, Giannoudis PV (2006). Growing bone and cartilage. The role of mesenchymal stem cells. J Bone Joint Surg Br.

[79] Gamradt SC, Lieberman JR (2004). Genetic modification of stem cells to enhance bone repair. Ann Biomed Eng.

[80] Dickson G, Buchanan F, Marsh D, Harkin-Jones E, Little U, McCaigue M (2007). Orthopaedic tissue engineering and bone regeneration. Technol Health Care.

[81] Luyten FP, Dell'Accio F, De Bari C (2001). Skeletal tissue engineering: opportunities and challenges. Best Pract Res Clin Rheumatol.

[82] Franceschi RT (2005). Biological approaches to bone regeneration by gene therapy. J Dent Res.

[83] Vinatier C, Guicheux J, Daculsi G, Layrolle P, Weiss P (2006). Cartilage and bone tissue engineering using hydrogels. Biomed mater Eng.

[84] Kofron MD, Laurencin CT (2006). Bone tissue engineering by gene delivery. Adv Drug Deliv Rev.

[85] Skripitz R, Aspenberg P (2004). Parathyroid hormone- a drug for orthopaedic surgery?. Acta Orthop Scand.

[86] Koester MC, Spindler KP (2006). Pharmacologic agents in fracture healing. Clin Sports Med.

[87] Aspenberg P (2005). Drugs and fracture repair. Acta Orthop.

[88] Aleksyniene R, Hvid I (2004). Parathyroid hormone- possible future drug for orthopaedic surgery. Medicina (Kaunas).

[89] Thomas T (2006). Intermittent parathyroid hormone therapy to increase bone formation. Joint Bone Spine.

